# Biomass and Extracts of *Metarhizium robertsii* MT008 as Potential Biopesticides for Controlling the Fruit Fly *Anastrepha obliqua*

**DOI:** 10.1007/s13744-025-01332-z

**Published:** 2025-11-27

**Authors:** Ginna Milena Quiroga-Cubides, Diana R. Vásquez Carreño, Diego Francisco Cortés-Rojas, Paola Emilia Cuartas-Otalora, Angela María Vargas-Berdugo, Buenaventura Monje Andrade, Edgar Herney Varón Devia, Eddy J. Bautista

**Affiliations:** 1 Corporación Colombiana de Investigación Agropecuaria-AGROSAVIA, Sede Central, Mosquera, Cundinamarca Colombia; 2 Corporación Colombiana de Investigación Agropecuaria-AGROSAVIA, Centro de Investigación Nataima, Espinal, Tolima Colombia

**Keywords:** Enthomopathogenic fungus, Dextrusin, Biological control, Submerged fermentation, Dual production, Mango

## Abstract

The fruit fly causes yield losses of 40 to 80% in various fruit crops, resulting in an approximate annual loss of USD 100 million in Colombia. *Anastrepha obliqua* is a significant pest for the mango production sector. Although biopesticides are promising alternatives for its control, there are currently no registered biopesticides derived from biomass or extracts of entomopathogenic fungi with the Colombian Agricultural Institute (ICA) for this purpose. This study demonstrates for the first time the potential of biomass and extracts from a native entomopathogenic fungus, *Metarhizium robertsii* MT008, cultivated through a liquid fermentation process, to effectively control both adult and preimaginal stages of *A. obliqua*. The optimal fermentation time was determined to be 5 days, producing the highest concentration of viable biomass (5.3 × 10^5^ CFU mL^−1^), along with 75.89 ppm of destruxin A and 147.58 ppm of destruxin B. The fungal extract caused 100% mortality in *A. obliqua* adults within 48 h. While the biomass without formulation achieved 100% mortality, a biomass prototype achieved nearly 90% mortality at doses of 0.10 and 0.20 mg of dry biomass per gram of vermiculite against the preimaginal stages of the fruit fly by 24 days post-inoculation under laboratory conditions. The results demonstrate strong potential for advancing biopesticides, as combining these bioactive agents could allow more effective control of *A. obliqua* populations in field conditions by causing mortality at two different stages of its life cycle.

## Introduction

The life cycle of fruit flies (Tephritidae) involves a stage in which the female pierces the fruit surface to oviposit, allowing the larvae to develop inside the fruit and feed internally. This internal damage is causing premature fruit drop and significant yield losses. Estimates indicate that fruit fly infestations can cause losses ranging from 40 to 80%, depending on the location, fruit variety, fruit fly species, and season (Dias et al. 2018; Martínez-Barrera et al. 2020). In Colombia, the economic impact is considerable, with losses associated with fruit flies estimated at US$100 million per year (DMH/LOFN 2025). According to the Colombian Agricultural Institute (ICA), “There are no fruit fly-free areas in Colombia, only areas with low prevalence” (Zambrano 2015). Similarly, fruit flies are among the most significant pests in global agriculture, causing major economic losses and acting as quarantine pests that restrict the export of fruits and vegetables. Their presence can lead to direct crop damage, reduced market value, and strict trade barriers, limiting access to international markets (Sciarretta et al. 2018; ICA 2021; Salazar-Mendoza et al. 2021; Opoku et al. 2025).

Two of the most important fruit fly species are *Ceratitis capitata* (Wiedemann), the Mediterranean fruit fly, and several native *Anastrepha* species, including *A. obliqua* (Macquart) and *A. ludens* (Wiedemann), which are recognized as quarantine pests (Presa-Parra et al. 2020; Salazar-Mendoza et al. 2021). *Anastrepha obliqua* is a polyphagous species widely distributed in the Neotropical region and is considered the main pest of mango (*Mangifera indica* L.) and *Spondias* spp. in Colombia and other Latin American countries (Hernández-Ortiz 1992; Osorio-Fajardo and Canal 2011; López et al. 2019; Aluja 2020).

Worldwide, the excessive and often indiscriminate use of chemical insecticides has led to resistance development in fruit fly populations against various active ingredients, including organophosphates, carbamates, pyrethroids, and Spinosad (Vontas et al. 2011; Sidana and Yadav 2022; Castells-Sierra et al. 2023; Majeed et al. 2025). Beyond resistance, the accumulation of pesticide residues in fruits is a critical public health and ecological concern. In Colombia, several studies have reported the presence of pesticide residues in mangoes intended for both export and domestic consumption markets (Vontas et al. 2011; Jaramillo-Colorado 2016; Balkan and Karaağaçlı 2023). The detectability and persistence of these residues frequently indicate overuse or non-targeted application, thereby contributing to bioaccumulation throughout the food chain. This accumulation not only jeopardizes food safety but also poses substantial ecological risks by disrupting soil microbiota, contaminating water sources, and causing imbalances within ecosystems. Furthermore, it results in soil contamination and harm to non-target organisms such as pollinators and natural predators. These concerns raise issues related to biodiversity and food safety in fruits, which consequently restrict their commercialization in international markets, as regulations in the European Union and the United States have established maximum residue limits (Hejazi et al. 2022; Balkan and Karaağaçlı 2023). These impacts worsen biodiversity loss and upset agroecosystem stability, highlighting the urgent need for more sustainable and ecologically friendly pest control options. In this context, biopesticides and their integration into integrated pest management (IPM) programs offer a viable, sustainable, and environmentally responsible alternative to reduce reliance on chemical pesticides while controlling pests (Bautista et al. 2018). Currently, only three biocontrol agents are officially registered locally for mango crops, all of which rely on pheromone- or attractant-based traps targeting adults, and none of them is based on entomopathogenic fungi (ICA 2025).

Entomopathogenic fungi such as *Beauveria bassiana* and *Metarhizium* spp. have demonstrated potential as effective biocontrol agents against fruit fly populations (Osorio-Fajardo and Canal 2011; Martínez-Barrera et al. 2020). These fungi, naturally occurring in the soil, can infect a broad range of insect pests, including various stages of *A. obliqua*. Laboratory and field studies have confirmed their pathogenicity to fruit fly larvae and adults using various application techniques, ranging from soil inoculation to auto-dissemination devices and baited traps (Ekesi et al. 2002; Dimbi et al. 2003; Sookar et al. 2008; Martínez-Barrera et al. 2020). Moreover, entomopathogenic fungi exert their biocontrol action through multiple mechanisms, including direct infection via resistant structures (conidia and blastospores) and the production of secondary metabolites and enzymes with insecticidal activity that could also be incorporated into IPM strategies on farms to improve pest control (Toledo-Hernández et al. 2018; Brito et al. 2019; Presa-Parra et al. 2020; Mejía et al. 2024; Angel-Ruiz et al. 2025). These also include cultural measures (such as fruit wrapping, sanitation, and early harvesting), the Male Annihilation Technique (MAT), the Bait Application Technique (BAT), and the Sterile Insect Technique (SIT) (Sciarretta et al. 2018; Aluja 2020; Hoskins et al. 2023; Opoku et al. 2025). Although such tools have shown considerable success when applied in area-wide integrated programs (Montoya and Cancino 2004; Toledo-Hernández et al. 2018; Enkerlin 2021), they are not always sufficient or cost-effective under field conditions, especially when pest pressure is high or when implementation lacks coordination.

Previous research conducted by Corporación Colombiana de Investigación Agropecuaria (AGROSAVIA) selected the fungus *Metarhizium robertsii* MT008 due to its capacity to induce mortality in the fruit fly *A. obliqua* preimaginal stages (third-instar larvae and/or pupae) through fungal resistant structures, and in adults via the action of its secondary metabolites. The compounds responsible for this insecticidal activity were identified as destruxins from groups E, D, A, and B (Lozano-Tovar et al. 2023). Based on this dual mode of action, it was hypothesized that both the *M. robertsii* MT008 biomass and its metabolites could serve as active components in the development of biological products targeting different life stages of the pest. Therefore, this study aimed to develop a submerged liquid fermentation process to produce fungal biomass and extracts from *M. robertsii* MT008 and to evaluate their potential as biopesticides for controlling *A. obliqua* in laboratory conditions.

## Materials and Methods

### Biological Material

The strain *M. robertsii* MT008 from the AGROSAVIA germplasm bank was used. It was obtained from the insect *Rhammatocerus schistocercoides* in the department of Meta (Villavicencio) and identified by the alpha 1 elongation factor (EF-1α) (Lozano-Tovar et al. 2023).

The larvae and adults of the fruit fly *A. obliqua* were obtained from the standardized laboratory culture of quarantine pests at the ICA regional office in Ibagué.

### Production of Biomass and Extracts from *M. robertsii* MT008

The dual production of biomass and destruxins was carried out in the liquid culture medium, coded MTM3, previously developed by AGROSAVIA. This medium contains an agro-industrial carbon source (6 g L^−1^), an organic nitrogen source (10 g L^−1^), and a basal medium (KH_2_PO_4_ = 1 g L^−1^, CaCl_2_−2H_2_O = 0.5 g L^−1^, MgSO_4_−7H_2_O = 0.5 g L^−1^, FeSO_4_−7H_2_O = 0.05 g L^−1^). The fermentation process started with the preparation of a pre-inoculum in a 250-mL Erlenmeyer flask, without baffles with an effective working volume of 100 mL of MTM3 medium and inoculated with 10 mL of a conidial suspension (1 × 10^6^ conidia mL^−1^) obtained from an *M. robertsii* MT008 growth on MAYP agar with a maximum age of 30 days. Pre-inoculum fermentation was carried out at 28 ± 0.5°C and 300 rpm for 4 days. A 7 mL volume of this pre-inoculum was used to inoculate the fermentation medium under the same operating conditions as the inoculum. Sampling was performed on days 4, 5, 6, and 7 with three replicates per sampling day.

On each sampling day, the following variables were measured: biomass concentration in dry weight (g L^−1^), destruxins A and B concentration (ppm), total protein content (ppm), structure concentrations (srt mL^−1^; submerged conidia mL^−1^; blastospores mL^−1^), and cell viability (CFU mL^−1^). Additionally, the biological activity of the extract on adult fruit flies was quantified every sampling day. pH was recorded at the beginning and end of fermentation. The biological activity of the biomass on *A. obliqua* preimaginal stages was assessed only on day 5. Ultrastructural analysis of the obtained structures was performed using scanning electron microscopy (SEM). All analytical techniques are described in the “Analytical techniques” and “Biological activity assays under laboratory conditions” sections.

A statistical analysis was conducted on biomass concentration, destruxins A and B levels, total protein content, structure concentrations, and cell viability. Initially, the data were subjected to normality testing (Shapiro–Wilk) and homoscedasticity testing (Bartlett). Subsequently, a one-way analysis of variance (ANOVA) was employed to assess statistical differences among the variables on days 4, 5, 6, and 7. Significant differences were further elucidated using Tukey’s HSD test at a 95% confidence level in Statistix® v8 (Analytical Software, Tallahassee, FL, USA). Data about the structures’ concentration and viability were subjected to decimal logarithm transformation before statistical analysis. The coefficient of variation (CV), calculated as the ratio of the standard deviation to the mean, was utilized as a parameter for the reproducibility of the variables.

### Biomass Formulation Prototypes for Controlling the Preimaginal Stages of the Fruit Fly* A. obliqua*

The biomass produced from the dual liquid fermentation process was formulated. To do this, the fermentation broth was centrifuged at 4500 rpm for 20 min to separate the biomass. Subsequent formulation trials focused on creating a solid, dispersible granule designed to control the insect’s preimaginal stages. 

### Analytical Techniques

To comprehensively evaluate the fermentation process of *M. robertsii* MT008, several variables of interest were measured. These encompassed (i) morphological and structural variables, such as the concentration of resistance structures, submerged conidia, and blastospores; (ii) growth-related variables, including biomass concentration and pH; (iii) physiological performance, assessed through cell viability; (iv) ultrastructural characteristics of biomass, analyzed by scanning electron microscopy; and (v) biochemical characterization through the quantification of secondary metabolites with insecticidal activity (destruxins A and B) and total protein content. Collectively, these variables facilitated the characterization of fungal development, viability, and metabolite production under the specified experimental conditions.

#### Structures concentration (srt mL^−1^; submerged conidia mL^−1^; blastospores mL^−1^)

Microscopic observation was performed on 0.5 mL samples to identify large structures (LS, > 10 µm; dispersed mycelium) and small structures (SS, < 10 µm; submerged conidia and blastospores). For large structures, 50-µL samples were observed using a light microscope with a ×5 objective. For small structures, the sample was treated with glass beads (*ø* = 0.5 cm) and vortexed for 1 min. The supernatant was collected, and the structures were quantified using a Neubauer counting chamber. Finally, total resistance structures were quantified (str mL^−1^).

#### Biomass concentration (g L^−1^)

Preweighed 50-mL centrifuge tubes were used to process 10 mL of culture broth, which was centrifuged at 15,000 rpm for 20 min. The recovered biomass was dried at 60 °C for 24 h to determine its dry weight. The results were expressed as the amount of dry biomass per liter of culture broth.

#### pH measurement

A 5-mL aliquot of culture broth was taken, and the pH was measured using a potentiometer (Consort C350, Germany).

#### Cell viability (CFU mL^−1^)

For viability assessment, triplicate samples of 1 mL of culture broth or 1 g of granulated prototypes were taken and subjected to serial decimal dilutions in 0.1% Tween solutions. Then, dilutions of 10^–3^, 10^–4^, and 10^–5^ were plated independently on MAYP agar by spreading 0.1 mL using a sterile microbiological spreader. Plates were incubated for 7 days at 25 ± 0.5°C and colony counts were recorded.

#### Ultrastructural analysis

Liquid samples were centrifuged at 15,000 rpm for 20 min. The supernatant was discarded, and the pellet was washed with 10 mL of sterile deionized water. The pellet was vortexed for 30 s and centrifuged again under the same conditions. This step was repeated two more times, and the resulting pellet was suspended in 10 mL of 2.5% v/v glutaraldehyde in phosphate buffer (pH 7) and fixed for 48 h. After fixation, samples were centrifuged and washed three times with sterile deionized water. Ultrastructural analysis of biomass was performed using a scanning electron microscope (Lyra 3, Tescan, USA).

#### Quantification of destruxin A and B (ppm) by liquid chromatography coupled to triple quadrupole mass spectrometry (LC-QqQ)

High purity standards were used for the analysis of destruxin A and B: destruxin A (≥ 98%, *Metarhizium anisopliae*, Sigma-Aldrich, D4921) and destruxin B (≥ 99.86%, MedChemExpress). The extraction process was carried out according to a previously described protocol with some modifications (Ríos-Moreno et al. 2017). Briefly, the samples from the extracts were centrifuged and filtered through 0.45 µm membranes. A 6-mL aliquot of the filtrate was collected and lyophilized. The dried extract was mixed with 1 mL of a blend of dichloromethane and ethyl acetate (equal parts), stirred at 110 rpm for 2.5 h at 28 °C, and then treated with ultrasound for 1 h. The liquid on top was collected and left to evaporate at room temperature for 5 h. The supernatant was collected and evaporated at room temperature for 5 h. The resulting residue was then dissolved in an acetonitrile:water solution (1:1, v/v) and injected into the chromatographic system.

Analysis was performed using a liquid chromatography system (Agilent Technologies 1260) coupled to a triple quadrupole mass spectrometer (LC-TQ 6470) equipped with an InfinityLab Poroshell C18 column (50 × 3.0 mm × 2.7 µm). A 1 µL injection volume was used with a mobile phase consisting of 0.1% (v/v) formic acid in Milli-Q water (phase A) and 0.1% (v/v) formic acid in acetonitrile (phase B), at a flow rate of 0.4 mL min^−1^ and a column temperature of 40°C. The gradient elution started at 5% phase B, increased to 95% over 15 min, held for 1 min, and re-equilibrated for 4 min. Data acquisition and processing were performed using MassHunter Qualitative Analysis version 10.0 (Agilent Technologies; exact release year not reported in official sources).

#### Total protein content (ppm)

Total protein quantification was performed on the extracts using the colorimetric method described by Bradford (1976), which is based on the interaction of the dye Coomassie Brilliant Blue G-250 with proteins in the sample (Bradford 1976). Hydrophobic and ionic interactions stabilize the anionic form of the dye, resulting in a visible color change that can be measured spectrophotometrically at 595 nm.

A calibration curve was generated using bovine serum albumin (BSA, Sigma-Aldrich, A7030) at concentrations of 0, 50, 100, 200, 300, 400, 500, 600, 700, and 800 mg L^−1^. For each assay, 20 µL of sample was mixed with 100 µL of Bradford reagent (Bio-Rad, Cat. 5,000,006), previously diluted 2:8 (reagent:water). Absorbance was measured using a BioTek Epoch spectrophotometer.

### Biological Activity Assays Under Laboratory Conditions

#### Biological Activity Assays for the Extracts of *M. robertsii* (MT008) Over Adults of* A. obliqua*

The insecticidal activity of the extracts was tested on adult *A. obliqua* fruit flies. Perforated containers holding 20 mL of the attractant Cebofrut© in the center were used, with an additional 20 mL of the extract applied to a gauze pad placed on the outer base of each container, depending on the treatment. The treatments were based on fermentation days: T1 was broth extract on day 4, T2 on day 7, T3 on day 5, and T4 on day 6. The control treatment used sterile distilled water instead of the extract to moisten the gauze. They were arranged in a completely randomized block design with three replicates.

The devices were individually suspended in the center of cages measuring 30 × 20 × 20 cm. Twenty healthy, newly emerged adult flies were released into each cage (Dimbi et al. 2003; Ouna 2010; Toledo-Hernández et al. 2018). The insects were kept and monitored at 30 °C with an average relative humidity of 60% and a photoperiod of approximately 12 h. Mortality was recorded at 48 h post-exposure.

#### Evaluation of *M. robertsii* MT008 Biomass and Formulation Prototype on *A. obliqua* Preimaginales Stages

The biomass and formulation prototypes of *M. robertsii* MT008 were evaluated against the preimaginal stages of *A. obliqua*. Each treatment, corresponding to different concentrations of fungal biomass or formulation prototype, was tested in triplicate. The treatments included T1, unformulated biomass; T2, prototype with 0.20 mg dry biomass per gram of vermiculite; T3, prototype with 0.10 mg dry biomass per gram of vermiculite; T4, prototype with 0.05 mg dry biomass per gram of vermiculite; T5, a control with conidia from solid-state fermentation at a concentration of 1 × 10⁶ conidia mL^−1^; and an absolute control group with no fungal biomass or prototype applied.

The biomass or the prototypes were dissolved and homogenized in a final volume of 30 mL of sterile distilled water supplemented with 0.05% Tween 20. Then, 10 mL of the resulting homogeneous mixture was added to three sterile 250-mL Erlenmeyer flasks, each containing 10 g of sterile vermiculite (Jaronski and Jackson 2008). For the absolute control treatment, the vermiculite was moistened only with 10 mL of sterile distilled water containing 0.05% Tween 20.

After homogenizing each treatment with vermiculite, 15 third-instar *A. obliqua* larvae were added to each flask. The flasks were then sealed with sterile Swiss voile and incubated under specified conditions: darkness, a temperature range of 22–25°C, and humidity above 75%. Three days post-inoculation, an initial check was performed to verify pupation in the absolute control group. Incubation continued for an additional 12 days until adults emerged. Once adults appeared at 24 days after inoculation, mortality among larvae, pupae, and adults was recorded and compared to the control group. All experiments were arranged in a completely randomized block design.

#### Mortality and Efficacy Calculations for the Various Treatments

Adult mortality was recorded at 48 h post-exposure, while preimaginal mortality was recorded at 24 days post-inoculation (standardized times in previous bioassays). Treatment efficacy was calculated using the Schneider–Orelli formula (Eq. 1) for uniform populations (Zar 1999).

1 $$\%Efficiency=\left[\left(A-B\right)/\left(100-B\right)\right]\times100$$

where *A* represents mortality in the treatment and *B* corresponds to mortality in the absolute control. Additionally, the efficacy data, which showed homogeneity of variances and normality according to the Bartlett (95%) and Shapiro–Wilk (95%) tests, respectively, were analyzed using an analysis of variance (ANOVA) and mean comparisons with the DMS or Tukey test (95%) using the Statistix® v8 (Analytical Software, Tallahassee, FL, USA).

## Results

### Production Dynamics of Resistant Structures During Liquid Fermentation Process

**Fig. 1:**
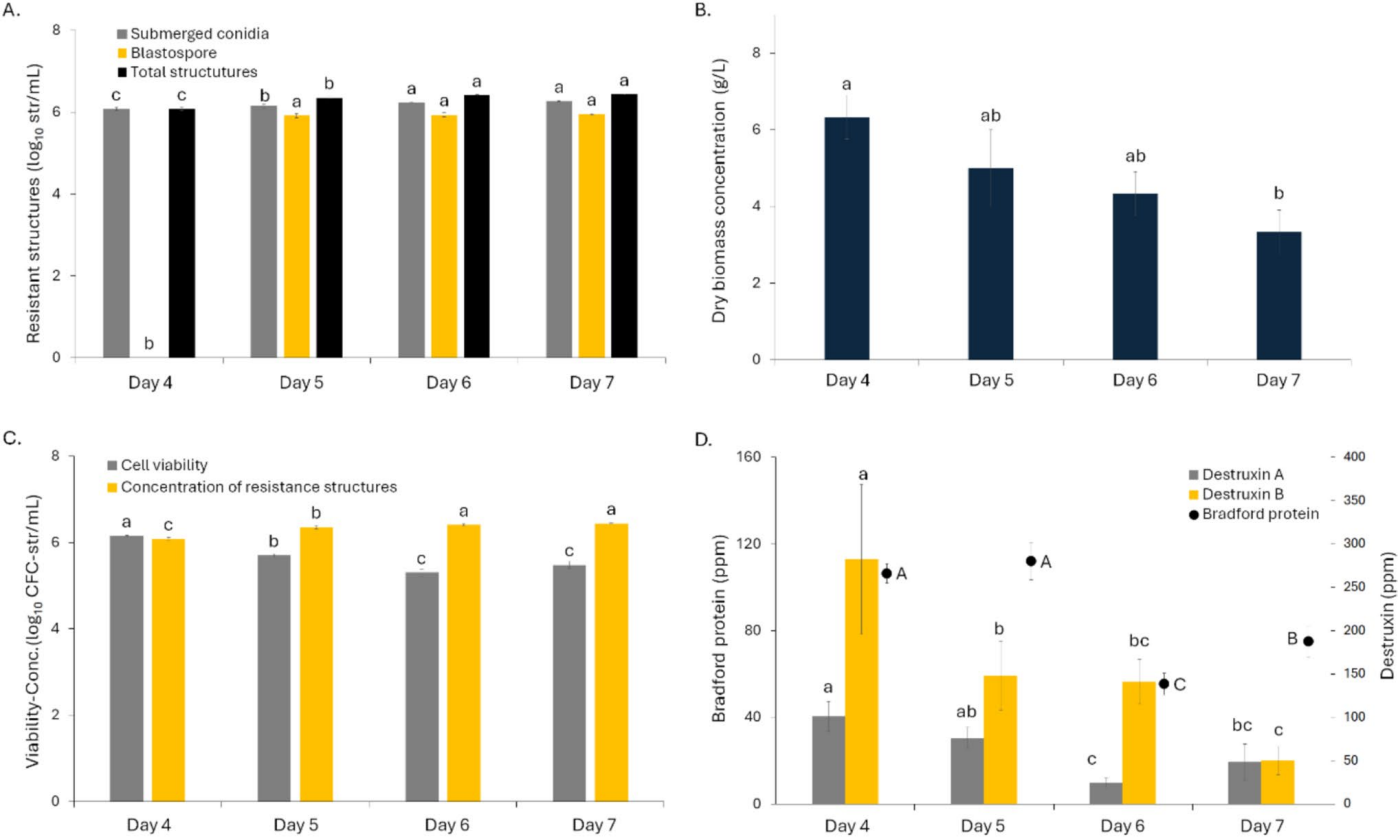
Monitoring resistant structures during submerged fermentation of *Metarhizium robertsii* MT008: **A** Concentration of resistant structures. **B** Dry biomass concentration. **C** Cellular viability and total structures concentration. **D** Total protein, destruxins A, and B concentrations. Treatments with different letters are significantly different according to Tukey’s test (95%)

Submerged conidia became visible from day 4, while blastospores were observed from day 6, suggesting that only dispersed mycelial forms were present before this point (Table 1). Regarding submerged conidia, the highest concentrations were recorded from day 6, reaching 1.5 × 10^6^ conidia mL^−1^ (Fig. 1A; *F*_3,32_ = 52.60, *p* < 0.0001). In contrast, the blastospore concentration remained significantly constant from day 5 onwards, ranging between 7 and 8 × 10^5^ blastospore mL^−1^ (Fig. 1A; *F*_3,32_ = 55,711, *p* < 0.0001). No large resistant structures such as microsclerotia were detected, although amorphous mycelial masses were observed (Table 1). Regarding the culture pH, it increased by more than two units during the first 4 days of fermentation, from 6.38 to 8.68, and then stabilized around pH 8 (Table 1).

**Table 1:**
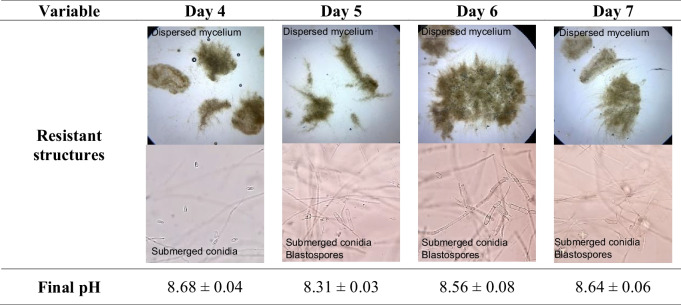
Monitoring resistant structures during submerged fermentation of *Metarhizium robertsii* MT008

The biomass concentration decreased significantly over time from an initial value of 6 g L^−1^ to a final value of 3.3 g L^−1^ (Fig. 1B; *F*_3,8_ = 3.98; *p* = 0.0524), and this decrease was correlated with a loss of cell viability (*F*_3,8_ = 66.2, *p* < 0.0001), indicating a potential compromise of cell integrity and functionality throughout the fermentation process (Fig. 1C). From day 5 onwards, a significant discrepancy between the total number of structures and cell viability was observed (*F*_1,4_ = 403, *p* < 0.0001), suggesting that a considerable proportion of the cells present were no longer viable.

Finally, the concentrations of resistant structures (conidia, blastospores, and total) and cell viability were found to be reproducible, with coefficients of variation below 2%. Process reproducibility is particularly relevant in biological systems where some variation is expected. A high variation (9% < CV > 20%) was observed for biomass concentration, which may be attributed to the dynamic nature of microbial growth and the inherent challenges in recovering fungal biomass, especially in cultures with abundant mycelial presence, as was the case here.

### Production of Secondary Metabolites

The concentrations of destruxins A and B showed statistically significant variations throughout the fermentation days, showing an inverse correlation between concentration and time (DTX A: *F*_3,8_ = 29.8, *p* = 0.0001; DTX B: *F*_3,8_ = 21.9, *p* = 0.0003; Fig. 1D). The CV of variation for the concentrations measured on each fermentation day ranged from 4 to 32%, irrespective of the sampling day. DTX A started at 101.12 ppm on day 4 and fell to 48.65 ppm by day 7. In contrast, DTX B exhibited significantly higher initial values, beginning at 282.14 ppm on day 4 and decreasing to 50.17 ppm by the end. Furthermore, DTX B maintained higher concentrations at every time compared to DTX A.

There were no statistically significant differences noted in total protein content between days 4 and 5; however, differences appeared on days 6 and 7 (*F*_3,8_ = 50.5, *p* < 0.0001; Fig. 1D). The CVs were found to be below 10%. An inverse correlation between total protein content and fermentation time was also observed from day 5 onwards. On day 4, the concentration was 106.37 ppm, which then slightly rose to 111.93 ppm on day 5, dropped to 55.26 ppm on day 6, and ultimately reached 74.89 ppm.

### Morphology of Resistance Structures Observed During Fermentation

**Fig. 2:**
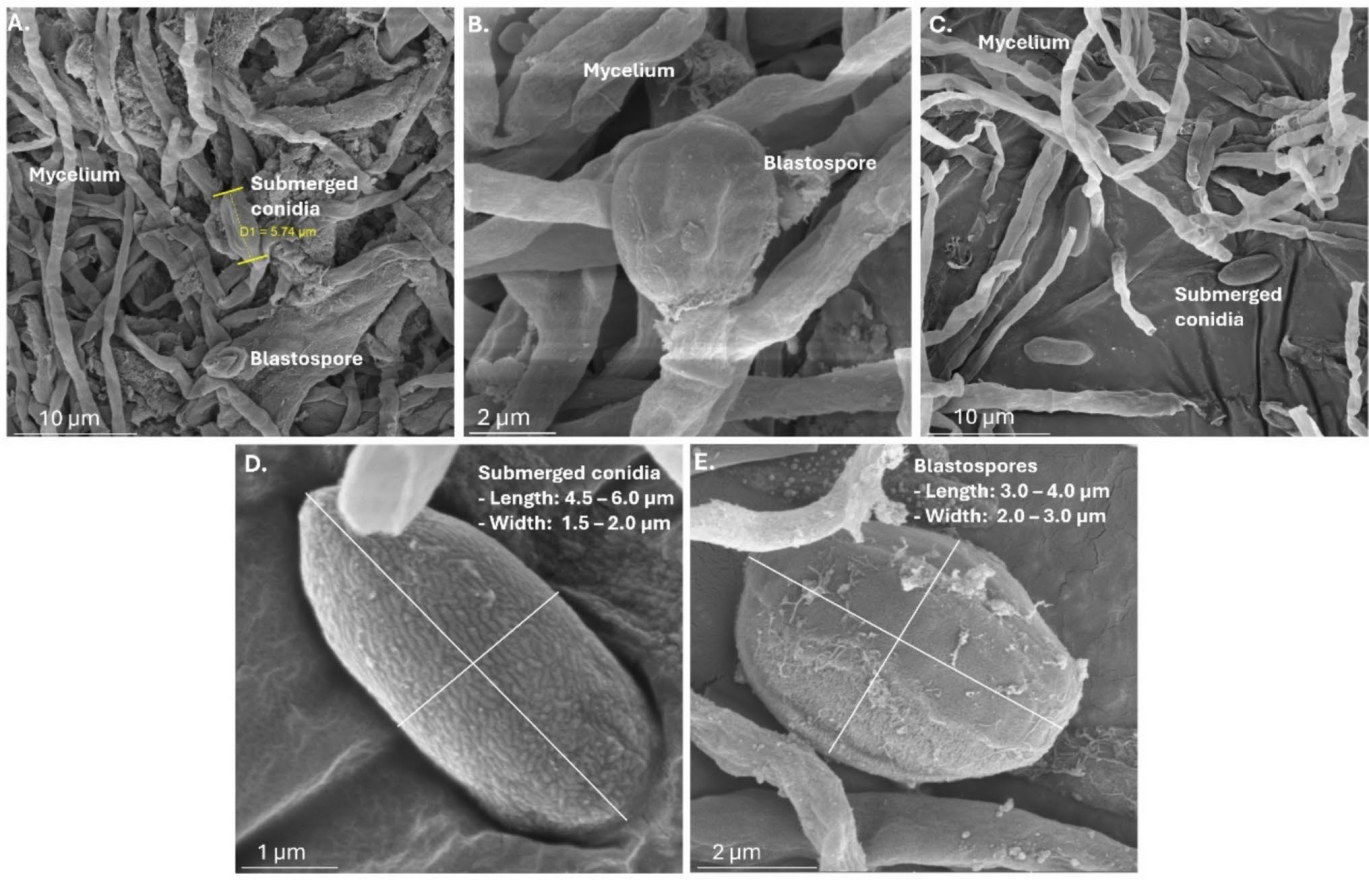
Ultrastructural analysis of *Metarhizium robertsii* MT008 resistance structures during submerged fermentation. Scanning electron microscopy (SEM) images illustrating the distinct morphological structures developed during submerged fermentation. **A** Mycelium network with submerged conidia and blastospores. **B** Detail of a blastospore emerging from hyphae. **C** Mycelium filaments and submerged conidia. **D** Detailed morphology and dimensions of submerged conidia. **E** Detailed morphology and dimensions of blastospores

Ultrastructural analysis using scanning electron microscopy (SEM) facilitated the characterization of various structures in the biomass of *M. robertsii* MT008. Within the mycelial masses, small formations such as submerged conidia and blastospores were identified (Fig. 2A–E). The conidia exhibited an elongated elliptical morphology (Fig. 2A, C, and D), with an average length of 5.25 µm and a width of 1.75 µm, while the blastospores appeared in a more spherical shape (Fig. 2B and E), measuring an average of 3.5 µm in length and 2.5 µm in width. The surface of the conidia was rough, whereas the blastospores had a smooth surface (Fig. 2D and E). Deposits of organic material were also noted, covering the structures and forming amorphous matrices within the mycelium (Fig. 2A and B).

### Biological Activity of Extracts, Biomass, and Biomass Prototypes at Different Doses

**Fig. 3:**
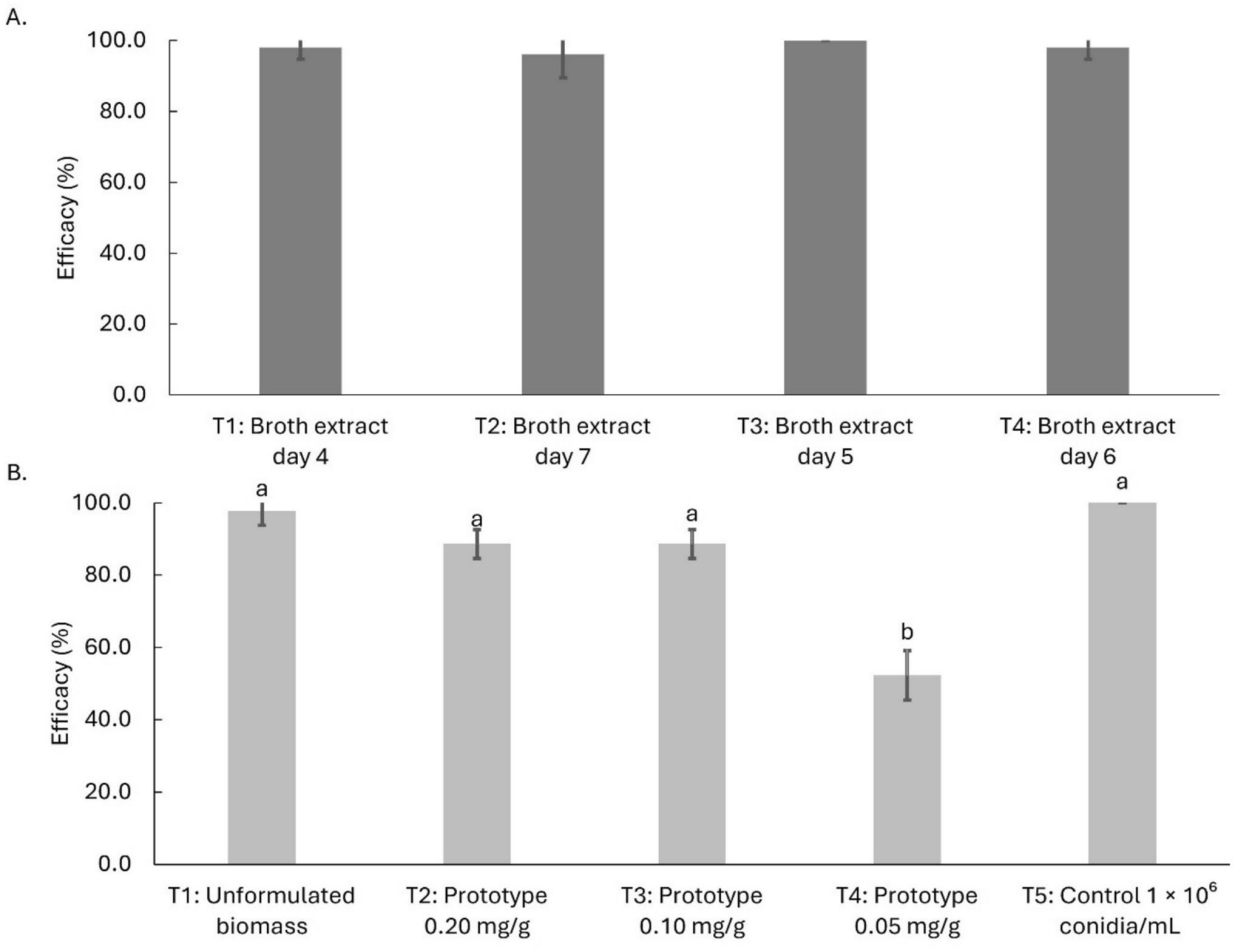
**A** Efficacy at 48 h of the extracts produced on different fermentation days on *Anastrepha obliqua* adults. **B** Efficacy at 24 days post-inoculation for *Metarhizium robertsii* MT008 biomass and prototypes against *Anastrepha obliqua* preimaginal stages. Treatments with different letters show significant differences according to Tukey’s mean comparison (95%)

The biological activity of extracts produced on days 4, 5, 6, and 7 of fermentation against *A. obliqua* adults showed that all extracts caused nearly 100% mortality, regardless of the fermentation day. No significant differences were observed between the different days, but each was significantly more effective than the absolute control, which had a 13.3% mortality (*F*_4,10_ = 201, *p* =  < 0.0001). Based on the efficacy calculated using corrected mortality from the absolute control (Fig. 3A), all treatments reached close to 100%, with no significant differences among them (*F*_3,8_ = 0.45, *p* = 0.7272). Notably, all replicates of day 5 of fermentation (treatment T3) achieved 100% efficacy within 48 h.

Fermentation should be stopped on day 5, when a balanced production of conidia and blastospores is observed, and total protein concentration reached its maximum. Therefore, the biomass obtained on day 5 was used to generate formulation prototypes at different doses, which were then tested for biological activity against preimaginal stages (Fig. 3B). Under the experimental conditions evaluated in this assay, preimaginal mortality of *A. obliqua* was significantly affected by the application of the various *M. robertsii* MT008 treatments, with mortality values ranging from 53.3% (T4) to 100% (T5). These values were statistically different from the mortality observed in absolute control, which was 2.2% (*F*_5,12_ = 253, *p* < 0.0001).

Based on efficacy, treatments T1, T2, T3, and T5 showed significantly higher efficacy compared to treatment T4, with values ranging from 88.6 to 100% (Fig. 3B). In contrast, treatment T4 had an efficacy of 52.3%. These results indicate that unformulated biomass of *M. robertsii* MT008 at a dose of 2.75 mg dry biomass g^−1^ vermiculite, along with the prototypes formulated at concentrations of 0.20 and 0.10 mg dry biomass g^−1^ vermiculite, demonstrates the highest biological activity against *A. obliqua* preimaginal stages. No adverse effects from the formulation were observed; however, a direct correlation with prototype concentration was evident. Furthermore, the results for treatments T1, T2, and T3 were comparable to the biological activity observed with aerial conidia of *M. robertsii* MT008, produced in Petri dishes and adjusted to a concentration of 1 × 10⁶ conidia mL^−1^ (T5)**.**

## Discussion

The dual-purpose submerged fermentation process developed in this study represents a novel approach for producing both fungal biomass and insecticidal metabolites with potential application in the biological control of *A. obliqua*. Both the unformulated extract and the biomass (whether formulated or unformulated) produced on the fifth day of fermentation exhibited high efficacy under laboratory conditions, inducing 100% mortality in adults and 88–100% mortality in preimaginal stages. These results either exceed or fall within the range reported in previous studies using *Beauveria bassiana* and *M. anisopliae* conidia, where mortality typically varied from 5 to 100% depending on strain and application method (Shaurub 2023). Previous Colombian studies have identified native strains of *B. bassiana* and *M. anisopliae* with promising pathogenicity against *A. obliqua* (Osorio-Fajardo and Canal 2011), and the compatibility of fungal agents with parasitoids has been documented (Martínez-Barrera et al. 2020). Nonetheless, this is the first report of *M. robertsii* achieving simultaneous control of both adult and immature stages of *A. obliqua*, significantly broadening the action spectrum of entomopathogenic fungi and confirming the value of native strains for locally adapted biocontrol strategies (Presa-Parra et al. 2020).

Day 5 was identified as the optimal harvest point due to the balanced production of conidia and blastospores, high protein content, and minimal differences between viability and resistance structure concentrations. Although blastospore yields were somewhat lower than values reported for other *Metarhizium* species (Jackson and Jaronski 2009), this variation likely reflects strain-specific biology and medium composition. Previous studies have shown that nutrient supplementation, aeration, agitation, and initial pH strongly influence propagule yields and that strains differ markedly in their responses to these parameters (Mascarin et al. 2015; Iwanicki et al. 2020; Bitencourt et al. 2023). The balanced propagule production and high destruxin output of MT008 suggest favorable genetic and physiological traits for biocontrol applications.

SEM analysis of the resistance structures revealed morphological distinctions between the conidia and blastospores of *M. robertsii* MT008, including differences in size and surface texture, with conidia exhibiting a rougher surface and blastospores appearing smoother, and are consistent with previous studies that describe morphological differences between conidia and blastospores in *Metarhizium* species. Moreover, the difference in surface texture (rough versus smooth) may influence adhesion to the host surface and resistance to environmental conditions, as previously suggested (Bernardo et al. 2018). Rough conidial surfaces, often ornamented with ridges or warts, can enhance mechanical interlocking with the insect cuticle and increase the available surface area for adhesive compounds, thereby improving spore adhesion and initial infection success (Holder and Keyhani 2005; Boucias and Pendland 2012). In contrast, the smoother surface of blastospores may reduce mechanical anchoring but has been associated with higher hydrophilicity, favoring germination under moist conditions, and facilitating rapid colonization once in contact with host tissues (Mascarin and Jaronski 2016). Additionally, surface morphology can influence environmental persistence. Conidia generally display greater tolerance to UV radiation, desiccation, and other abiotic stressors (Inglis et al. 2001), whereas blastospores are metabolically more active and capable of producing extracellular mucilage that aids in adhesion under humid conditions (Holder et al. 2007). These differences suggest that the dual production of conidia and blastospores, as observed on day 5 of fermentation, may confer complementary ecological advantages such as ensuring persistence and dissemination in drier environments, and enhancing infection efficiency in humid microhabitats. On the other hand, the amorphous matrices within the mycelium suggest the presence of metabolic exudates or extracellular matrix components that may play a role in protecting, adhesion and cuticle penetration in *Metarhizium* spp. (Wang and St Leger 2007; Leemon and Jonsson 2012). These structures could play a role in protecting fungal propagules from environmental stress and in facilitating cooperative infection processes. Therefore, the morphological and surface characteristics observed in this study are not merely taxonomic traits but may directly contribute to the high virulence observed against both adult and immature stages of *A. obliqua*.

The destruxin concentrations obtained here represent the highest recorded under submerged fermentation of *Metarhizium* spp., surpassing previously reported yields such as 126.9 mg L⁻^1^ of DTX A and 47.5 mg L⁻^1^ of DTX B for *M. brunneum* BIPESCO 5 (Taibon et al. 2014) and up to 163 mg L⁻^1^ for *M. anisopliae* ARSEF-2735 with colloidal chitin supplementation (Opoku et al. 2025). While strain identity plays an important role, fermentation conditions also strongly influence destruxin biosynthesis. In this study, cultures tended to become slightly alkaline, a condition previously associated with enhanced destruxin expression. DTX B in particular peaks at approximately pH 9, whereas acidic conditions below pH 5 inhibit production (Liu et al. 2007; Liu and Tzeng 2012). Nitrogen availability also exerts a major influence, with balanced C/N ratios supporting high yields (Park et al. 2018). Thus, the elevated destruxin levels recorded here likely reflect both favorable strain physiology and advantageous fermentation dynamics. These results demonstrate that the cultivation strategy employed was highly effective for promoting secondary metabolite biosynthesis, providing a valuable platform for producing metabolite-enriched formulations.

In Colombia, registered biopesticides for fruit fly control rely on pheromone- or attractant-based traps targeting adults (ICA 2025). No products are currently available that can simultaneously suppress both adult and immature stages of *A. obliqua*. Internationally, the only comparable product is Campaign® (*M. anisopliae* spores), which is registered in parts of Africa for soil and auto-dissemination applications (International Centre of Insect Physiology and Ecology [icipe], 2025). In contrast, this study proposes two complementary bioproducts: biomass-derived formulations acting primarily against larvae and pupae through contact and extract-based formulations targeting adults via ingestion. Such a dual approach allows mortality induction at multiple stages of the insect life cycle. This stage-specific strategy is particularly relevant for neotropical mango orchards, where alternating wet and dry seasons shape pest dynamics. Biomass-based products could be deployed during rainy periods to exploit favorable soil moisture conditions for larval and pupal infection, while extract-based formulations could be applied during the dry season to target adult populations. Together, these complementary products support a seasonally tailored, integrated management plan for *A. obliqua*. Field efficacy trials, stability studies, and compatibility assessments with other IPM tools such as sterile insect technique (SIT), male annihilation technique (MAT), and parasitoid release will be crucial for advancing operational use.

## Conclusion

This study shows that the native *M. robertsii* MT008 can be effectively grown using a submerged fermentation method that produces both viable fungal biomass and extracts containing dextrusins. Both products demonstrated strong insecticidal activity against various developmental stages of *A. obliqua*, exceeding previously reported levels for related *Metarhizium* species. The simultaneous production of conidia, blastospores, and secondary metabolites emphasizes the strain’s versatility and potential for creating stage-specific biopesticides. By offering a dual-action tool that targets both adult and immature stages, this approach fills an important gap in fruit fly control in the Neotropics. It provides a solid basis for developing integrated pest management strategies tailored to local agroecological conditions.

## Data Availability

The data that support the findings of this study are available from the corresponding author upon reasonable request. Please contact ebautista@agrosavia.co to request access.
